# Actigraphy in studies on insomnia: Worth the effort?

**DOI:** 10.1111/jsr.13750

**Published:** 2022-10-11

**Authors:** Lara Rösler, Glenn van der Lande, Jeanne Leerssen, Roy Cox, Jennifer R. Ramautar, Eus J. W. van Someren

**Affiliations:** ^1^ Department of Sleep and Cognition Netherlands Institute for Neuroscience Amsterdam The Netherlands; ^2^ Departments of Integrative Neurophysiology, Center for Neurogenomics and Cognitive Research, Amsterdam Neuroscience VU University Amsterdam The Netherlands; ^3^ Department of Child and Adolescent Psychiatry, Amsterdam UMC University of Amsterdam Amsterdam The Netherlands; ^4^ Departments of Integrative Neurophysiology and Psychiatry, Center for Neurogenomics and Cognitive Research, Amsterdam UMC, Amsterdam Neuroscience VU University Amsterdam The Netherlands

**Keywords:** actigraphy, insomnia, non‐parametric circadian rhythm analysis, sleep, sleep scoring

## Abstract

In the past decades, actigraphy has emerged as a promising, cost‐effective, and easy‐to‐use tool for ambulatory sleep recording. Polysomnography (PSG) validation studies showed that actigraphic sleep estimates fare relatively well in healthy sleepers. Additionally, round‐the‐clock actigraphy recording has been used to study circadian rhythms in various populations. To this date, however, there is little evidence that the diagnosis, monitoring, or treatment of insomnia can significantly benefit from actigraphy recordings. Using a case–control design, we therefore critically examined whether mean or within‐subject variability of actigraphy sleep estimates or circadian patterns add to the understanding of sleep complaints in insomnia*.* We acquired actigraphy recordings and sleep diaries of 37 controls and 167 patients with varying degrees of insomnia severity for up to 9 consecutive days in their home environment. Additionally, the participants spent one night in the laboratory, where actigraphy was recorded alongside PSG to check whether sleep, in principle, is well estimated. Despite moderate to strong agreement between actigraphy and PSG sleep scoring in the laboratory, ambulatory actigraphic estimates of average sleep and circadian rhythm variables failed to successfully differentiate patients with insomnia from controls in the home environment. Only total sleep time differed between the groups. Additionally, within‐subject variability of sleep efficiency and wake after sleep onset was higher in patients. Insomnia research may therefore benefit from shifting attention from average sleep variables to day‐to‐day variability or from the development of non‐motor home‐assessed indicators of sleep quality.

## 1 INTRODUCTION

Actigraphy has been used to estimate sleep for multiple decades. As humans move significantly less during sleep than while awake, actigraphs, commonly placed on the wrist, measure acceleration to allow inferences about sleep. Validation studies, comparing actigraphy sleep scoring to the gold‐standard polysomnography (PSG), have shown that actigraphy fares relatively well in healthy populations, as well as in certain sleep disorders (Sadeh, 2011). Patients with insomnia struggle with sleep initiation or maintenance and the non‐restorative nights have an impact on their daytime functioning (Daley et al., 2009). To date, the clinical diagnosis of insomnia is based predominantly on subjective reports (Sateia, 2014), despite a growing literature of physiological substrates of the disorder (Riemann et al., 2010, 2017). While PSG studies have shown little avail in detecting the stark subjective differences between patients with insomnia and healthy controls in a single laboratory night (Riemann et al., 2017), actigraphy offers a relatively cheap and easily implementable alternative to monitor sleep in insomnia over multiple nights. However, patients with insomnia may lie still for prolonged periods of time while continuing to remain awake, rendering actigraphy assessment of sleep difficult (Hauri & Wisbey, 1992; Paquet et al., 2007). Still, various studies have allegedly validated actigraphy against PSG for use in insomnia (Kushida et al., 2001; Lichstein et al., 2006; Marino et al., 2013; Mccall & Mccall, 2012), although with inconsistent results (Sivertsen et al., 2006; Taibi et al., 2013; Vallières & Morin, 2003). Crucially, the majority of validation studies compared actigraphy against PSG during a single night in the laboratory (Kushida et al., 2001; Lichstein et al., 2006; Marino et al., 2013; Mccall & Mccall, 2012), while researchers largely use actigraphy for the monitoring of multiple nights of sleep in the home environment (Sadeh, 2011).

The few available studies on ambulatory actigraphy in insomnia have reported inconsistent results. The agreement between self‐reported sleep and actigraphy measures was shown to be low for community dwelling older adults with insomnia symptoms (Scarlett et al., 2021), mirroring earlier findings on actigraphic sleep estimates and self‐reported insomnia symptoms (Chen et al., 2015). Williams et al. (2018), however, showed that ambulatory actigraphy reliably estimated sleep parameters in young adults with insomnia compared with ambulatory PSG. Similarly, another study in adults with insomnia showed that multiple nights of ambulatory actigraphy yielded similar sleep estimates to a single laboratory PSG night (Withrow et al., 2019). However, a third of the participants failed to provide sufficient bedtime markers, suggesting that study compliance might frequently stand in the way of optimal data analysis. Neither study included control participants, thereby precluding conclusions about the sensitivity of ambulatory actigraphy to distinguish patients with insomnia from controls.

Actigraphy has further been suggested in the International Classification of Sleep Disorders manual as a tool to monitor alterations in circadian rhythms in insomnia (Sateia, 2014). Here, activity counts are not scored as sleep or wakefulness, but rather the full 24 h of activity patterns over multiple days are used to determine diurnal rhythm variables such as interdaily stability (IS), intradaily variability (IV), and epochs of maximum and minimum activity (van Someren et al., 1999). This method has been helpful in differentiating patients suffering from delayed sleep phase onset syndrome (DSPS) from patients with insomnia (Ancoli‐Israel et al., 2003). Accordingly, Dagan et al. (1998) showed that multiple days of actigraphy home recordings can aid the clinical assessment of DSPS, in line with another study that showed that wrist actigraphy patterns can distinguish people with DSPS from normal sleepers (Minors et al., 1996). Within patients with insomnia, however, there is little evidence for circadian rhythm disturbances, potentially rendering the monitoring of circadian rhythms with actigraphy superfluous in this group. While van Veen et al. (2010) reported an attenuated 24 h amplitude of the rest–activity pattern and lower interdaily stability of adults with insomnia comorbid with ADHD, no differences in inter‐ or intradaily rhythm variables were found in children with ADHD and insomnia (van der Heijden et al., 2005). The few other studies published on diurnal rhythm variables in insomnia also failed to find differences between patients and controls (Kim, Lim, Kwon, & Lee, 2020; Natale et al., 2009), with the actual numbers of null results possibly being much larger due to the file drawer problem (Rosenthal, 1979).

The objective of the current study was to investigate to what extent actigraphy can aid our understanding of insomnia. To this end, we acquired actigraphy recordings and sleep diaries for up to 9 days in the home environment of patients with insomnia and controls, as well as one night of simultaneous recordings of actigraphy and polysomnography in the laboratory. Based on the inconsistent results in the literature, we did not expect to find differences in actigraphy‐derived sleep parameters between patients and controls in their home environments, despite a likely high agreement between polysomnography and actigraphy in the laboratory.

## 2 METHODS

### 2.1 Participants

Participants with insomnia and controls without sleep complaints were recruited through the Netherlands Sleep Registry (www.slaapregister.nl), advertisements, and media to join a longitudinal study on insomnia between November 2018 and September 2019 (Leerssen et al., 2020). We only considered applicants between 18 and 70 years of age. After application, we conducted an interview by phone to verify that the participants suspected of having insomnia, based on a screening including the Insomnia Severity Index (ISI; Morin et al., 2011), met the general inclusion and insomnia criteria, and were not currently undergoing treatment for insomnia. Healthy controls were considered for participation if they had an ISI score below 10 and did not meet any DSM‐5 criteria for insomnia disorder. Exclusion criteria for both patients and controls relevant for the present analyses were: (1) a current diagnosis of major depressive disorder or anxiety disorder, (2) current treatment with antidepressant medication, (3) current CBT‐I treatment, (4) moderate to severe sleep apnea syndrome, moderate to severe restless legs syndrome or severe periodic limb movement disorder, (5) self‐reported diagnosis of a severe neurological or psychiatric disorder, (6) self‐reported severe physical or mental impairment due to stroke, or traumatic head injury, and (7) current shift work (see Leerssen et al., 2020 for a complete list of exclusion criteria). The use of sleep medication was permitted and monitored. A total of 18 patients included in the analyses were taking prescription hypnotics (alprazolam, temazepam, diazepam, lorazepam, oxazepam, quetiapine, zolpidem, or zopiclon). With the exception of WASO variability, the results did not change when these 18 patients were excluded from the analyses (see Tables S1 and S2 for results without medicated patients). In total, actigraphy data were available for 209 participants but due to drop‐out (*n* = 1) and issues with actigraphy data quality (*n* = 4), 204 participants (146 females) were included in the final sample (see Table 1 for further sample characteristics). Of the 204 participants, 167 met the ICSD3 and DSM‐5 criteria for Insomnia Disorder.

**TABLE 1:** Sample characteristics

	Patients with insomnia (*n* = 167)	Controls (*n* = 37)	*p*
Male/female	48/119	10/27	0.850
Age (years)	49.2 ± 0.9	48.4 ± 2.2	0.741
Insomnia severity (ISI)	15.8 ± 0.3	3.0 ± 0.6	<0.001
Depressive symptom severity (IDS‐SR)	17.9 ± 0.6	5.35 ± 4.7	<0.001
Anxiety severity (BAI)	7.06 ± 0.5	2.22 ± 0.4	<0.001
Subjective sleep estimates			
Total sleep time (min)	358 ± 4.8	438 ± 9.5	<0.001
Sleep efficiency (%)	75.6 ± 0.9	90.6 ± 1.8	<0.001
Sleep onset (min)	31.5 ± 1.8	12.1 ± 3.6	<0.001
Wake after sleep onset (min)	48.2 ± 1.5	14.7 ± 3.0	<0.001
Sleep opportunity window (min)	473 ± 3.7	484 ± 7.3	0.164

*Note*: Subjective sleep estimates based on sleep diaries (mean ± standard error).

### 2.2 Procedure

Ambulatory recordings were part of a larger study (Leerssen et al., 2020) approved by the Medical Ethics Committee of the VU University Medical Centre (NL63139.029.17) and obtained once the participants had provided written informed consent. The participants received ActTrust 2 actigraphs (Condor Instruments, São Paulo, Brazil) during their first laboratory visit, with detailed instructions. For up to nine consecutive days and nights, wrist movement was monitored with a sampling rate of 25 Hz. On average, the participants had 6.78 ± 2.3 24 h windows of data available (6.73 ± 2.30 for patients and 7.03 ± 2.35 for controls). The participants filled out sleep diaries daily throughout this time period (Consensus Sleep Diary [Carney et al., 2012]). Upon completion of the ambulatory assessment, the participants spent one night in the laboratory for simultaneous actigraphy and polysomnography recording. Prior to the ambulatory assessment, the participants filled out multiple questionnaires including a demographics questionnaire.

### 2.3 Data processing

#### 2.3.1 Sleep diaries

Consensus sleep diary variables were calculated daily per participant and included the following: sleep opportunity window (SOW, calculated as the time period between closing one's eyes and final awakening when no further attempt to sleep was made), total sleep time (TST), sleep efficiency (TST divided by SOW), sleep onset latency (i.e., minutes to fall asleep), and wake after sleep onset (WASO; i.e., minutes awake after sleep onset including time attempting to sleep after final awakening).

#### 2.3.2 Actigraphy sleep parameters

Activity counts assessed in 30 s epochs were initially inspected visually and compared against subjectively documented bedtimes. If visible periods of non‐wear time of at least 2 h fell into the documented bedtime window, these nights were discarded from the analyses. We subsequently obtained epoch‐wise sleep versus wake estimates using the open‐source package *PyActigraphy* (Hammad et al., 2021), implementing a device‐tuned version of the Cole‐Kripke algorithm provided by actigraph manufacturer Condor Instruments. Epochs were analysed within the sleep diary‐derived SOW for each night, defined as the time between closing one's eyes and no longer trying to sleep after waking up for the last time. We subsequently calculated total sleep time (TST, calculated as the sum of all epochs scored as sleep during the SOW), sleep efficiency (SE, calculated as TST divided by SOW), sleep onset latency (SOL, calculated as the time between closing one's eyes and the first sleep epoch) and wake after sleep onset (WASO, calculated as the sum of all wake epochs after the first sleep epoch and prior to the end of the SOW) for the available nights in the home environment.

#### 2.3.3 Actigraphy nonparametric circadian rhythm variables

For the determination of nonparametric circadian rhythm variables (NPCRA), activity counts were again initially inspected visually. Periods of non‐wear larger than 4 h at any time of the day were marked and extended to 24 h periods to exclude them from analysis. This approach prevents differential bias on NPCRA features that would result from exclusion of nocturnal restful versus diurnal active hours. Subsequently, four different variables were calculated for the recording period in the home environment: Interdaily stability (IS) describing the stability of the rest–activity rhythm across multiple days; intradaily variability (IV) quantifying the fragmentation of the rest–activity pattern; the least active 5 h (L5); and the 10 h with maximal activity (M10).

Interdaily stability is calculated as the 24 h value from the chi‐square periodogram, normalised for the number of data. It can be easily calculated as the ratio between the variance of the average 24 h pattern around the mean and the overall variance:

$$\text{IS}=\frac{n\sum_{h=1}^{p}{\left({\bar{x}}_{h}-\bar{x}\right)}^{2}}{p\sum_{i=1}^{n}{\left(x_{i}-\bar{x}\right)}^{2}}$$

where n is the total number of data, p is the number of data per day, ${\bar{x}}_{h}$ are the hourly means, $\bar{x}$ is the mean of all data, and $x_{i}$ represents the individual data points. The IS varies between zero for Gaussian noise and one for perfect IS. However, values around zero are reached only for lengthy data sets.

The intradaily variability is calculated as the ratio of the mean squares of the difference between all successive hours (first derivative) and the mean squares around the grand mean (overall variance):

$$\mathrm{IV}=\frac{n\sum_{i=2}^{n}{\left(x_{i}-x_{i-1}\right)}^{2}}{\left(n-1\right)\sum_{i=1}^{n}{\left(x_{i}-\bar{x}\right)}^{2}}$$

The IV values reach near zero for a perfect sine wave; it is about 2 for Gaussian noise and may even be higher when a definite ultradian component with a period of 2 h is present.

M10 and L5 averages are calculated on the level of minutes, by averaging the values of the most active 10 h and least active 5 h per day across days.

#### 2.3.4 Polysomnography

Polysomnography was collected using a 256‐channel LTM HydroCel Geodesic Sensor Net referenced to Cz, and additional bipolar physiological signals including chin electromyography, sampled at 1000 Hz by a Net Amps 300 amplifier (Electrical Geodesic Inc.). Sleep scoring was performed in 30 s epochs using standard channels and filters following the American Academy of Sleep Medicine (AASM) manual (Berry et al., 2017). Polysomnography data were available for 192 participants. For an overview of standard sleep architecture estimates in our sample see Table S3.

In order to match the variables available for actigraphy, PSG sleep variables were also calculated during the diary‐defined SOW (the time between closing one's eyes and giving up on sleeping in the morning). Accordingly, TST was defined as the sum of all sleep epochs during the SOW, SE was defined as TST divided by SOW, SOL was calculated as the first sleep epoch (N1, N2, N3, or R) following eyes closed and WASO was defined as the sum of all wake epochs after the first sleep epoch and prior to the end of the SOW.

#### 2.3.5 Statistical analyses

Actigraphy‐derived sleep parameters, as well as sleep‐diary defined sleep variables were evaluated with linear mixed effect models for multi‐day measurements within each participant, using the *lme4* package (Bates et al., 2014) in R (version 4.1.1). For both actigraphy and sleep diary, four mixed models each evaluated group differences with respect to TST, SE, SOL, and WASO. For sleep diaries, we performed an additional mixed model analysis investigating potential group differences in the SOW. All models included insomnia diagnosis as a regressor, age, and sex as covariates and subject identity as a random intercept. The significance of predictors was estimated with an analysis of variance table using Satterwaithe's method in the R‐package lmerTest (Kuznetsova et al., 2017).

Given previous reports on between‐night instability of sleep in insomnia (Buysse et al., 2010; van Someren, 2007; Wohlgemuth et al., 1999), we additionally tested whether within‐subject night‐to‐night variability in sleep diaries and actigraphic estimates of TST, SE, SOL, and WASO differed between people with insomnia and controls. Variability was defined as the standard deviation of each sleep parameter per participant. These differences were then evaluated using single‐level linear models, including insomnia diagnosis as predictor, as well as age and sex as covariates. To account for possible effects of a varying number of nights per participant (overall mean = 6.78, SD = 2.34), we included the number of nights as an additional covariate.

Group differences in the four nonparametric circadian rhythm variables (IS, IV, L5, M10) were evaluated using single‐level linear models as multiday recordings give one value per participant. Models again included insomnia diagnosis as the predictor and age and sex as the covariates.

Comparisons between polysomnography and actigraphy sleep scoring in the laboratory were performed using Cohen's kappa to evaluate epoch‐wise agreement. To this end, available PSG sleep scoring was binarised into sleep versus wakefulness. Cohen's kappa was first calculated per participant and then averaged across the entire sample. We additionally performed Spearman's rank correlations to estimate the agreement of PSG and actigraphy for the four sleep variables TST, SE, SOL, and WASO.

We considered an effect significant if the value of *p* <0.05. Analyses scripts are publicly available on OSF (https://osf.io/t2n6k/).

## 3 RESULTS

### 3.1 Subjective sleep

Linear mixed models on sleep diaries revealed that patients with insomnia reported significantly shorter TST (*β* = −79.98 min, 95% CI [−100.0; −59.9], *p* = <0.001), lower SE (*β* = −14.97%, 95% CI [−18.8; −11.2], *p* = <0.001), longer WASO (*β* = 32.94 min, 95% CI [22.6; 43.3], *p* = <0.001), and longer SOL (*β* = 19.20 min, 95% CI [11.6; 26.8], *p* = <0.001). The SOW did not differ reliably in people with insomnia (*β* = −11.13 min, 95% CI [−26.6; 4.3], *p* = 0.162). Subjective sleep parameters were also more variable on a night‐to‐night basis in patients with insomnia (all *p* < 0.001). Table 1 shows averages and standard deviations of subjectively estimated sleep.

### 3.2 Average actigraphic sleep estimates

**FIGURE 1:**
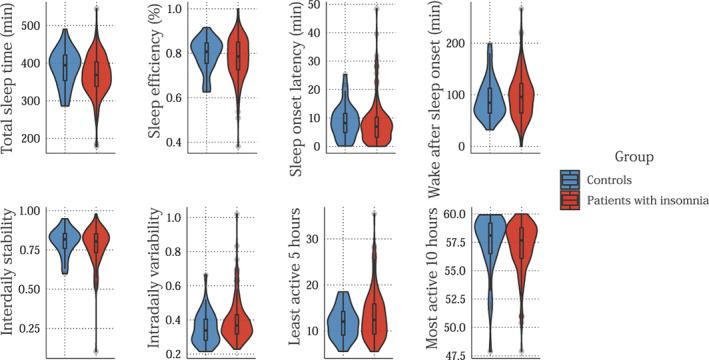
Violin plots and box plots showing the distribution of individual scores on actigraphy‐derived sleep variables (upper panels) and circadian rhythm variables (lower panels) for controls without sleep problems (blue) and patients with insomnia (red)

Patients with insomnia had a significantly shorter TST (*β* = −21.23 min, 95% CI [−30.3; −3.1], *p* = 0.023). However, no differences between patients and controls were found for SE (*β* = −1.87%, 95% CI [−4.9; 1.3], *p* = 0.244), SOL (*β* = 0.23 min, 95% CI [−2.2; 2.6], *p* = 0.853), nor WASO (*β* = 6.11 min, 95% CI [−8.7; 20.9], *p* = 0.422). Figure 1 and Table 2 give an overview of actigraphic sleep estimate averages and standard deviations.

**TABLE 2:** Ambulatory actigraphy results

	Patients with insomnia	Controls	*p*
Actigraphy sleep estimates			
Total sleep time (min)	368 ± 4.3	389 ± 8.6	0.023
Sleep efficiency (%)	78.0 ± 0.7	79.9 ± 1.4	0.244
Sleep onset (min)	8.29 ± 0.6	8.06 ± 1.2	0.853
Wake after sleep onset (min)	96.3 ± 3.5	90.2 ± 7.0	0.422
Total sleep time variability	49.9 ± 1.9	45.4 ± 3.7	0.259
Sleep efficiency variability	0.06 ± 0.0	0.04 ± 0.0	0.007
Sleep onset variability	8.31 ± 0.5	8.77 ± 1.1	0.691
Wake after sleep onset variability	29.6 ± 1.5	23.2 ± 3.0	0.049
Circadian rhythm variables			
Interdaily stability	0.78 ± 0.01	0.81 ± 0.02	0.214
Intradaily variability	0.40 ± 0.01	0.36 ± 0.02	0.052
Least active 5 h	13.7 ± 0.41	12.1 ± 0.83	0.090
Most active 10 h	57.2 ± 0.19	57.5 ± 0.38	0.478

*Note*: Actigraphy‐derived sleep variables, within‐subject variability of these sleep variables, as well as circadian rhythm variables (mean ± standard error).

### 3.3 Night‐to‐night variability in actigraphic sleep estimates

Investigations of within‐subject night‐to‐night variability of sleep variables revealed more variable SE in patients with insomnia (*β* = 1.30%, 95% CI [0.3; 2.2], *p* = 0.007), as well as more variable WASO (*β* = 6.39 min, 95% [0.0; 12.7], *p* = 0.049). Neither TST within‐subject variability (*β* = 4.52 min, 95% CI [−3.4; 12.4], *p* = 0.259), nor SOL within‐subject variability (*β* = −0.46 min, 95% CI [−2.8; 1.8], *p* = 0.691) differed between groups.

### 3.4 Nonparametric circadian rhythm variables

Patients with insomnia did not differ from controls on interdaily stability (*β* = −0.02, 95% CI [−0.1; 0.0], *p* = 0.214), nor on intradaily variability (*β* = 0.04, 95% CI [0.0; 0.1], *p* = 0.053), L5 (*β* = 1.53, 95% CI [−0.2; 3.3], *p* = 0.090), and M10 (*β* = −0.29, 95% CI [1.1; 0.5], *p* = 0.478). Table 2 provides an overview of circadian rhythm variable averages and standard deviations.

### 3.5 Laboratory‐based agreement of actigraphy with PSG

Epoch‐wise agreement between actigraphy and PSG sleep scoring (sleep vs wakefulness) was moderate across the entire sample as demonstrated by an average Cohen's kappa of 0.44 (SD = 0.17). Separate group investigations revealed similar results – patients with insomnia showed an average Cohen's kappa of 0.44 (SD = 0.17), while control participants had an average Cohen's kappa of 0.44 (SD = 0.19). Spearman rank correlation tests further revealed a strong correlation for PSG‐ and actigraphy‐estimated TST (*r*_s_ = 0.75, *p* < 0.001), a moderate association for SE (*r*_s_ = 0.48, *p* < 0.001), as well as WASO (*r*_s_ = 0.54, *p* < 0.001), and a significant but modest association for SOL (*r*_s_ = 0.31, *p* < 0.001) in patients with insomnia. The results were similar for controls, where we observed a strong correlation for TST (*r*_s_ = 0.64, *p* < 0.001), a moderate association for WASO (*r*_s_ = 0.48, *p* < 0.001), as well significant but modest associations for SE (*r*_s_ = 0.38, *p* < 0.001) and SOL (*r*_s_ = 0.38, *p* < 0.001).

## 4 DISCUSSION

Actigraphy has gained increasing popularity for home sleep monitoring in insomnia research. However, while various studies have demonstrated high agreement between single‐night laboratory polysomnography and actigraphy in insomnia, very few studies have systematically investigated the sensitivity of ambulatory actigraphy to actually capture the complaints experienced by people suffering from insomnia. In a sample size representative of clinical studies, we validated actigraphic estimates of sleep against PSG in the laboratory, and recorded multiple nights of actigraphy in the home environment to evaluate the sensitivity of actigraphic sleep estimates to distinguish insomnia from good sleep. Despite substantial agreement between actigraphy and PSG sleep scoring during the laboratory night, we found that the vast majority of ambulatory actigraphy sleep variables did not help to differentiate patients with insomnia from controls. Patients differed from controls only in showing a shorter average sleep duration and a higher within‐subject night‐to‐night variability in both sleep efficiency and wake after sleep onset. The effect sizes were small, respectively *d* = −0.15, *d* = 0.18, and *d* = 0.14 and these variables might therefore be of limited use in assisting the insomnia diagnosis of individual participants. Combined, the findings raise questions about the role and utility of actigraphy in insomnia research. Possibly, shifting the focus from estimating average sleep variables to investigating sleep variability will be more conclusive.

Multiple day and night actigraphy recordings in participant's home environment did not reveal substantial differences in motor‐activity‐based sleep estimates between patients with insomnia and controls. While sleep duration was significantly reduced by an average of 21 min in patients with insomnia, this was considerably less than the subjectively estimated 80 min discrepancy between groups. In view of the observed internight variability, this modest group difference additionally suggests that actigraphy‐derived sleep duration might not be suitable to diagnose single individuals. Arguably, the most defining characteristic of sleep quality is not overall duration, but the balance between achieved sleep and time spent trying to achieve and maintain sleep, best captured by sleep efficiency. Neither for sleep efficiency, nor for wake after sleep onset or sleep onset latency, was actigraphy sensitive enough to distinguish patients with insomnia from controls. These findings are in line with previous observations in studies with smaller sample sizes. Despite showing high agreement between ambulatory actigraphy and polysomnography in insomnia, Sánchez‐Ortuño et al. (2010) found that patients differed from controls only on onset latency. In another study, none of the actigraphic sleep estimates differentiated patients from controls (Buysse et al., 2010). It is not unlikely that a much larger number of null results have been left unreported (Rosenthal, 1979). In recent years, vast efforts have been undertaken to improve the quality of actigraphy sleep estimates for insomnia research (Angelova et al., 2020; Rösler et al., 2022; te Lindert et al., 2020). In a retrospective study of more than 400 participants, Natale et al. (2014) concluded that actigraphy satisfactorily differentiates patients with insomnia from controls, when the correct quantitative actigraphic criteria for the used device are applied. In their study, however, patients and controls stemmed from two distinct databases with distinct study protocols, contesting the assumption that the two groups were fit for a valid comparison. Certainly, a vast range of available actigraphic devices, algorithms and analysis techniques challenge the conclusions any single one study can draw. Nevertheless, considering the small number of studies investigating the sensitivity of ambulatory actigraphy in insomnia in combination with the present null findings, ambulatory actigraphy might not be as useful a tool for insomnia monitoring as commonly assumed.

We further failed to observe any significant differences in circadian rhythm variables between patients with insomnia and controls. Rest–activity patterns in insomnia were not more fragmented, nor less stable across days than in our control group. In the 2003 paper on the use of actigraphy in the study of sleep, Ancoli‐Israel et al. (2003) suggested that actigraphy can be used to assess circadian rhythm disturbances secondary to other sleep disorders or psychiatric conditions. Since then, strikingly few papers have been published on rest–activity patterns in insomnia. Kerkhof and van Vianen (1999) showed that individual differences among patients with insomnia with respect to sleep duration and sleep onset latency were associated with phase differences in the 24 h temperature rhythm. However, multiple studies failed to find general differences in circadian rhythm variables between patients with insomnia and controls (Kim, Lim, Kwon, & Lee, 2020; Kim, Lim, Suh, & Lee, 2020; Natale et al., 2014; van der Heijden et al., 2005). Considering the heterogeneity of insomnia (Benjamins et al., 2017), it is nonetheless conceivable that certain subtypes of insomnia might suffer from circadian rhythm disturbances. However, the current evidence suggests that alterations in rest‐activity patterns are not a general feature of the majority of people suffering from insomnia.

Our actigraphy analyses revealed more variable sleep efficiency and wake after sleep onset in patients with insomnia, which is also reflected in the subjective sleep diary entries. These results parallel observations made by Buysse et al. (2010), who also showed increased variability in these sleep variables in insomnia. While our effect sizes are small (*d* = 0.18 and *d* = 0.14 respectively), they suggest that day‐to‐day variability in sleep might be more pertinent to identifying insomnia and potential treatment responses. Our study investigated possible group differences both in variability of round‐the‐clock recorded activity (IS), as well as in activity recorded specifically only during the nocturnal sleep window. This combination allows us to conclude that zooming in on night‐to‐night variability may be more sensitive than assessing variability in the overall 24 h activity pattern. Previous work already stressed that sleep estimates become more reliable with an increasing number of nights (van Someren, 2007; Wohlgemuth et al., 1999). For within‐subject variability, in particular, future studies should therefore ensure a recording length of sufficient duration.

The current results call for modesty with respect to the usefulness of actigraphy in insomnia research, but some limitations also need to be considered. Although our sample included a relatively large number of patients with insomnia compared with the majority of previously published validation studies (Williams et al., 2018; Withrow et al., 2019), our control group only included 37 participants. For optimal statistical power, group sizes should ideally be comparable. Ambulatory actigraphy analyses in our study also yielded relatively low sleep estimates for our control group, raising potential concerns whether these participants represented true controls. Importantly, during recruitment all control participants underwent rigorous screening to detect and exclude people with sleep complaints, reflected in the high sleep efficiency (90%) subsequently obtained via sleep diaries during our acquisition phase. Additionally, average sleep efficiency below 85% have previously been reported for the same device in healthy good sleepers (Mantua et al., 2016), confirming that subjective good sleep and poorer objective sleep efficiency are not irreconcilable. A final concern about our study design is that we relied on subjective sleep diaries to define the SOW used for actigraphy sleep scoring. Possibly, actigraphy would achieve better discriminatory results if more objective bedtime markers (e.g., lights‐out or bed pressure sensors) would be implemented.

Taken together, our data suggest that current state‐of‐the‐art ambulatory actigraphy might not be sufficiently sensitive to reliably discriminate patients with insomnia from good sleepers when comparing average sleep estimates. As the scarce literature on rest–activity patterns in insomnia suggested, we also did not find evidence for general circadian rhythm disturbances in patients with insomnia. We are therefore left to question to what extent multiple day actigraphy recordings can actually contribute to our understanding of insomnia. While actigraphy is a non‐invasive assessment, the purchase of devices, distribution and handling, wearing by patients, reading out and analysing data etc., all in all takes a lot of investment, time and effort. Our results suggest that insomnia research might benefit from shifting efforts to the investigation of day‐to‐day variability of sleep estimates, as previously advised by others (Angelova et al., 2020; Bei et al., 2016; Buysse et al., 2010; Lemola et al., 2013; Sánchez‐Ortuño & Edinger, 2012). Alternatively, or additionally, future efforts should focus on the development of non‐motor home‐assessed indicators of sleep quality, for example either directly using alternative biosignals, or indirectly by assessment of overnight adaptation of distress and arousal (van Someren, 2021).

## AUTHOR CONTRIBUTIONS

Lara Rösler, Jennifer R. Ramautar, and Eus J. W. van Someren designed the study. Glenn van der Lande, Jeanne Leerssen, and Jennifer R. Ramautar acquired the data. Lara Rösler and Roy Cox analysed the data. Eus J. W. van Someren supervised the study and Lara Rösler prepared the initial draft with input from all the authors. All authors provided critical reviews of the manuscript.

## CONFLICT OF INTEREST

No potential conflict of interest is reported by the authors.

## Supporting information


**TABLE S1** Sample characteristics for non‐medicated patients and controls. Subjective sleep estimates based on sleep diaries (mean ± standard error)
**TABLE S2** Ambulatory actigraphy results for non‐medicated patients and controls. Actigraphy‐derived sleep variables, within‐subject variability of these sleep variables, as well as circadian rhythm variables (mean ± standard error)
**TABLE S3** Polysomnography sleep estimates (mean ± standard error). Sleep period was defined as sleep onset to final awakening. All sleep estimates were subsequently calculated within the sleep period or as a percentage thereof

## Data Availability

The data that support the findings of this study are available on request from the corresponding author. The data are not publicly available due to privacy or ethical restrictions.
