# Supplementary material for: Actigraphy in studies on insomnia: Worth the effort?
Source: J Sleep Res. 2022 Oct 11;32(1):e13750. doi: 10.1111/jsr.13750 (PMC10078209; doi:10.1111/jsr.13750)
Supplement: Supplementary file 1 — TABLE S1 Sample characteristics for non‐medicated patients and controls. Subjective sleep estimates based on sleep diaries (mean ± standard error) TABLE S2 Ambulatory actigraphy results for non‐medicated patients and controls. Actigraphy‐derived sleep variables, within‐subject variability of these sleep variables, as well as circadian rhythm variables (mean ± standard error) TABLE S3 Polysomnography sleep estimates (mean ± standard error). Sleep period was defined as sleep onset to final awakening. All sleep estimates were subsequently calculated within the sleep period or as a percentage thereof [file JSR-32-0-s001.docx]

**Supplementary Material**

**Actigraphy in studies on insomnia: worth the effort?**

Lara Rösler^1^, Glenn van der Lande^1^, Jeanne Leerssen^1,2^, Roy Cox^1^, Jennifer R. Ramautar^3^, Eus J. W. van Someren^1,4^

^1^Netherlands Institute for Neuroscience, Department of Sleep and Cognition, Amsterdam, The Netherlands

^2^Departments of Integrative Neurophysiology, Center for Neurogenomics and Cognitive Research, Amsterdam Neuroscience, VU University, Amsterdam, The Netherlands

^3^ Department of Child and Adolescent Psychiatry, Amsterdam UMC, University of Amsterdam, Amsterdam, Netherlands

^4^Departments of Integrative Neurophysiology and Psychiatry, Center for Neurogenomics and Cognitive Research, VU University, Amsterdam UMC, Amsterdam Neuroscience, Amsterdam, The Netherlands

Corresponding author:

Lara Rösler

Meibergdreef 47, 1105 BA Amsterdam, The Netherlands

[l.rosler@nin.knaw.nl](mailto:l.rosler@nin.knaw.nl)

**Results without medicated patients**

As 18 of the 167 patients with Insomnia Disorder were taking prescription hypnotics which might affect sleep quality and architecture, we have repeated our analyses without the inclusion of these participants. Apart from WASO variability, none of the reported results changed in direction or significance level (see Supplementary Tables S1 and S2 below).

|  | Patients with insomnia  (*n* = 149) | Controls  (*n* = 37) | *p* |
| --- | --- | --- | --- |
| Male/female | 43/106 | 10/27 | .986 |
| Age (years) | 49.0 ± 1.0 | 48.4 ± 2.2 | .808 |
| Insomnia severity (ISI) | 15.7 ± 0.3 | 3.0 ± 0.6 | <.001 |
| Depressive symptom severity (IDS-SR) | 17.8 ± 0.6 | 5.35 ± 4.7 | <.001 |
| Anxiety severity (BAI) | 7.07± 0.5 | 2.22 ± 0.4 | <.001 |
| ***Subjective sleep estimates*** |  |  |  |
| Total sleep time (min) | 363 ± 4.7 | 440 ± 8.8 | <.001 |
| Sleep efficiency (%) | 76.9 ± 0.9 | 90.9 ± 1.6 | <.001 |
| Sleep onset (min) | 29.0 ± 1.7 | 12.3 ± 3.2 | <.001 |
| Wake after sleep onset (min) | 46.0 ± 2.5 | 14.4 ± 4.7 | <.001 |
| Sleep opportunity window (min) | 472 ± 4.0 | 485 ± 7.4 | .105 |

**Table S1. Sample characteristics for non-medicated patients and controls**. Subjective sleep estimates based on sleep diaries (mean ± standard error).

|  | Patients with insomnia | Controls | *p* |
| --- | --- | --- | --- |
| ***Actigraphy sleep estimates*** |  |  |  |
| Total sleep time (min) | 368 ± 4.5 | 390 ± 8.4 | .016 |
| Sleep efficiency (%) | 78.2 ± 0.8 | 79.9 ± 1.5 | .302 |
| Sleep onset (min) | 7.93 ± 0.5 | 8.13 ± 1.2 | .856 |
| Wake after sleep onset (min) | 95.6 ± 3.8 | 90.2 ± 7.2 | .488 |
| Total sleep time variability | 50.6 ± 2.0 | 45.9 ± 3.8 | .258 |
| Sleep efficiency variability | 0.06 ± 0.0 | 0.04 ± 0.0 | .009 |
| Sleep onset variability | 8.26 ± 0.6 | 8.79 ± 1.1 | .640 |
| Wake after sleep onset variability | 29.2 ± 1.6 | 23.2 ± 3.1 | .075 |
| ***Circadian rhythm variables*** |  |  |  |
| Interdaily stability | 0.78 ± 0.01 | 0.81 ± 0.02 | .223 |
| Intradaily variability | 0.40 ± 0.01 | 0.36 ± 0.02 | .061 |
| Least active 5 hours | 13.7 ± 0.45 | 12.1 ± 0.86 | .093 |
| Most active 10 hours | 57.3 ± 0.19 | 57.4 ± 0.38 | .704 |
|  |  |  |  |

**Table S2. Ambulatory actigraphy results for non-medicated patients and controls.** Actigraphy-derived sleep variables, within-subject variability of these sleep variables, as well as circadian rhythm variables (mean ± standard error).

|  | Patients with insomnia  (*n* = 157) | Controls  (*n* = 35) | *p* |
| --- | --- | --- | --- |
| Sleep period (min) | 448 ± 4.2 | 476 ± 8.9 | .006 |
| Total sleep time (min) | 389 ± 4.9 | 426 ± 10.4 | <.001 |
| Sleep efficiency (%) | 86.5 ± 0.7 | 89.7 ± 1.4 | .045 |
| Sleep onset (min) | 14.2 ± 1.1 | 12.6 ± 2.2 | .490 |
| Wake after sleep onset (min) | 59.8 ± 2.9 | 49.1 ± 6.2 | .123 |
| N1 (min) | 31.5 ± 1.5 | 31.5 ± 3.2 | .999 |
| N2 (min) | 205 ± 3.9 | 209 ± 8.2 | .643 |
| N3 (min) | 81.4 ± 2.6 | 95.1 ± 5.6 | .028 |
| REM (min) | 70.9 ± 2.4 | 90.8 ± 5.1 | <.001 |
| N1 (% of sleep period) | 7.0 ± 0.3 | 6.5 ± 0.6 | .499 |
| N2 (% of sleep period) | 45.5 ± 0.7 | 44.1 ± 1.5 | .416 |
| N3 (% of sleep period) | 18.4 ± 0.6 | 20.1 ± 1.3 | .210 |
| REM (% of sleep period) | 15.7 ± 0.5 | 19.0 ± 1.0 | .003 |

**Table S3.** PSG sleep estimates (mean ± standard error). Sleep period was defined as sleep onset to final awakening. All sleep estimates were subsequently calculated within the sleep period or as a percentage thereof.
